# Evolution of quality of life, anxiety, and depression over time in patients with an abdominal aortic aneurysm approaching the surgical threshold

**DOI:** 10.1093/bjsopen/zrae150

**Published:** 2025-01-10

**Authors:** Alexander Vanmaele, Petros Branidis, Maria Karamanidou, Elke Bouwens, Sanne E Hoeks, Jorg L de Bruin, Sander ten Raa, K Martijn Akkerhuis, Felix van Lier, Ricardo P J Budde, Bram Fioole, Hence J M Verhagen, Eric Boersma, Isabella Kardys

**Affiliations:** Department of Cardiology, Thorax Centre, Cardiovascular Institute, Erasmus MC, Rotterdam, The Netherlands; Department of Vascular Surgery, Erasmus MC, Rotterdam, The Netherlands; Department of Cardiology, Thorax Centre, Cardiovascular Institute, Erasmus MC, Rotterdam, The Netherlands; Department of Vascular Surgery, Erasmus MC, Rotterdam, The Netherlands; Department of Cardiology, Thorax Centre, Cardiovascular Institute, Erasmus MC, Rotterdam, The Netherlands; Department of Vascular Surgery, Erasmus MC, Rotterdam, The Netherlands; Department of Cardiology, Thorax Centre, Cardiovascular Institute, Erasmus MC, Rotterdam, The Netherlands; Department of Vascular Surgery, Erasmus MC, Rotterdam, The Netherlands; Department of Anaesthesiology, Erasmus MC, Rotterdam, The Netherlands; Department of Anaesthesiology, Erasmus MC, Rotterdam, The Netherlands; Department of Vascular Surgery, Erasmus MC, Rotterdam, The Netherlands; Department of Vascular Surgery, Erasmus MC, Rotterdam, The Netherlands; Department of Cardiology, Thorax Centre, Cardiovascular Institute, Erasmus MC, Rotterdam, The Netherlands; Department of Anaesthesiology, Erasmus MC, Rotterdam, The Netherlands; Department of Radiology and Nuclear Medicine, Erasmus MC, Rotterdam, The Netherlands; Department of Vascular Surgery, Maasstad Hospital, Rotterdam, The Netherlands; Department of Vascular Surgery, Erasmus MC, Rotterdam, The Netherlands; Department of Cardiology, Thorax Centre, Cardiovascular Institute, Erasmus MC, Rotterdam, The Netherlands; Department of Cardiology, Thorax Centre, Cardiovascular Institute, Erasmus MC, Rotterdam, The Netherlands

## Abstract

**Background:**

Contrary to the impact of screening, the effect of long-term surveillance on the quality of life of patients with an abdominal aortic aneurysm is not well known. Therefore, the aim of this study was to describe patient-reported outcomes of patients with an abdominal aortic aneurysm approaching the surgical threshold.

**Methods:**

This multicentre, observational cohort study included patients with an abdominal aortic aneurysm with a maximum aneurysm diameter of greater than or equal to 40 mm. The EuroQol five-dimension five-level questionnaire (range −0.446 to 1, minimal clinically important difference 0.071), the Hospital Anxiety and Depression Scale questionnaire (0–21 points/subscale, minimal clinically important difference 1.7 points), and the short version of the Patient Health Questionnaire (0–6 points) were mailed to patients with an abdominal aortic aneurysm at baseline and after 1 and 2 years or until abdominal aortic aneurysm surgery/death. Linear mixed-effects models were used to describe the evolution of patient-reported outcomes over time and investigate changes attributable to clinical characteristics.

**Results:**

In total, 291 to 294 responses to each questionnaire were available from 124 patients with an abdominal aortic aneurysm, of whom 34 underwent surgery during follow-up. The mean health-related quality of life and anxiety and depression scores over time were 0.781 (95% c.i. 0.749 to 0.814), 4.4 points (95% c.i. 3.9 to 4.9), and 4.6 points (95% c.i. 4.0 to 5.2) respectively. Anxiety and depression scores decreased in patients who underwent surgery with a mean of 2.8 (95% c.i. 1.1 to 4.6) and 2.0 (95% c.i. 0.4 to 3.6) points/year respectively, compared with patients who had not had surgery. Considering the minimal clinically important difference, patients with a primary education alone, compared with a secondary education, had higher or increasing anxiety and depression scores. Patients with a first-degree relative with an abdominal aortic aneurysm had a higher risk of clinical anxiety.

**Conclusion:**

Although health-related quality of life, anxiety, and depression remain stable over time on average, anxiety and depression decrease in patients approaching surgery. Patients with a family history of abdominal aortic aneurysm or a primary education alone experience more anxiety and/or depression and thus might benefit from a tailored approach during surveillance.

## Teaser

Contrary to the impact of screening, the effect of long-term surveillance on the quality of life of patients with an abdominal aortic aneurysm is not well known. Therefore, by means of repeatedly collected questionnaires (the EuroQol 5-dimension 5-level questionnaire, the Hospital Anxiety and Depression Scale questionnaire, and the short version of the Patient Health Questionnaire) over time in a prospective cohort of patients with an abdominal aortic aneurysm, it was found that patients with an abdominal aortic aneurysm under surveillance have a stable, slightly lower health-related quality of life than previously reported in the general population, but comparable anxiety and depression scores, which decrease as the date of surgery approaches. Additionally, patients with a first-degree relative with an abdominal aortic aneurysm or a primary education alone experience more anxiety and/or depression, and might therefore benefit from a tailored approach throughout surveillance.

## Introduction

Patients diagnosed with an abdominal aortic aneurysm (AAA) undergo periodic surveillance, until open or endovascular repair is proposed to prevent rupture^1,2^. Most AAAs are small upon detection, leaving patients under surveillance for several years, with a majority of patients never reaching the surgical threshold during their lifetime^3^.

AAA screening programmes have demonstrated benefits in prevention of rupture and reducing AAA-related deaths^4^. Whereas qualitative studies report contrasting patient experiences regarding an AAA diagnosis through screening programmes^5,6^, quantitative measures of patient experience do not indicate any impact on a patient’s quality of life (QoL)^6^. Nevertheless, screening does increase the number of patients with a diagnosis of a small AAA; these patients undergo periodic surveillance until they qualify for surgery^4,7^. Although several studies investigated the QoL of patients during screening, most reports are limited to the first year of surveillance and thus might not reflect the long-term implications of an AAA diagnosis or its relationship to AAA progression^8^. Second, reports that do focus on longer time intervals after screening are limited to health-related QoL alone^9,10^; anxiety and depression are insufficiently explored in the more general questionnaires used by these reports. Lastly, the interval surrounding surgery might be an exceptionally vulnerable interval in terms of patient experience, given the substantial AAA size and the foresight of qualifying for surgery in the near future^11^.

Given the lack of knowledge on patient-reported outcomes in patients with an AAA approaching the surgical threshold, the aim of this study was to assess the evolution of health-related QoL, anxiety, and depression, and their determinants, in patients with an AAA under periodic surveillance.

## Methods

### Study design

The BIOMArCS-AAA study is a prospective observational cohort study, carried out at the Erasmus MC and Maasstad Hospital in the Netherlands, aimed at identifying blood biomarkers associated with AAA. A full study design has been published previously^12^ and is briefly described below and displayed in *Fig. S1*. The present study was focused on a longitudinal cohort within the BIOMArCS-AAA study of patients with an AAA with a maximum aneurysm diameter of greater than or equal to 40 mm and not currently scheduled for intervention. Patients with thoracic, isolated iliac, or non-degenerative aneurysms, with end-stage renal disease, with a linguistic barrier, or unlikely to appear/be able to complete the follow-up were excluded. The study was carried out in accordance with the Declaration of Helsinki and ethics approval was obtained from the medical ethics committee at the Erasmus MC. Written informed consent was acquired. The study was registered at ClinicalTrials.gov (NCT03703947).

Patient education in both hospitals consisted of a combination of patient information leaflets and verbal information, left to the discretion of the (small group of) treating vascular surgeons.

After a baseline visit, participants were followed over 2 years or until AAA surgery/death within this time frame. QoL questionnaires were sent yearly to participants through the mail to be completed at home and returned in a franked envelope. In the case of unreturned questionnaires, patients were encouraged at study visits to complete these documents, again, at home and return them by post. For the present analyses, all patients with an AAA with at least one questionnaire were included, using all available response data.

### Questionnaires

Patient-reported outcomes were evaluated through three questionnaires (the EuroQol 5-dimension 5-level (EQ-5D-5L) questionnaire, the Hospital Anxiety and Depression Scale (HADS) questionnaire, and the short version of the Patient Health Questionnaire (PHQ-2)). AAA-specific questionnaires were not used, given the lack of validated instruments at the time of the inclusion interval of the study^13–15^. The EQ-5D-5L questionnaire is a general tool that assesses various dimensions of QoL, including mobility, self-care, regular activities, pain/discomfort, and anxiety/depression. A country-specific index score can be calculated, ranging for the Netherlands between −0.446 and 1, where an index score of 1 represents perfect health^16,17^. The choice of this questionnaire to quantify health-related QoL was based on the current lack of studies employing this tool in patients with an AAA under surveillance, contrasted by its broad use in patients with an AAA undergoing aneurysm repair and extensive reporting in different patient populations, and based on its validation in the Dutch general population. The minimal clinically important difference (MCID) for the EQ-5D-5L questionnaire has previously been proposed to be 0.071 for patients with cardiovascular disease^18^. The questionnaire ends with a visual analogue scale (VAS), ranging from 0 to 100. The HADS questionnaire focuses on the core components of anxiety and depression, specifically targeting mental health without including physical aspects. Given its broad use and previous validation, this questionnaire was chosen to specifically elucidate the evolution of anxiety and depression in patients with an AAA under surveillance (both subscales (anxiety and depression) consist of 7 questions, with scores ranging from 0 to 3 for each item, with a total score up to 21 per subscale)^19^. Its MCID for patients with cardiovascular disease has been established to be 1.7 points^20^. The PHQ-2 is a screening tool for major depressive disorder (patients rate the frequency of both depressed mood and anhedonia on a 4-level scale (0 to 3), with a total score up to 6)^21^. This third questionnaire was chosen to concisely validate the findings of the HADS tool.

The EQ-5D-5L and HADS scores were treated as continuous variables. Additionally, the HADS scores were dichotomized using a cut-off of greater than or equal to 8 to represent possible clinically relevant anxiety or depressive signs^19^. The PHQ-2 was dichotomized using a cut-off of greater than or equal to 3 to indicate the presence of major depressive symptoms^21^. Missing values were imputed when at least one question per questionnaire was answered and imputation was stratified per moment in time. A single imputed data set was created using multiple imputation by chained equations^22^.

### Statistical analysis

Continuous variables are presented as mean(s.d.) or median (interquartile range (i.q.r.)), depending on normality (determined by looking at histograms and QQ plots, as well as by the use of the Shapiro–Wilk test). Categorical variables are presented as *n* (%).

Linear mixed-effects (LME) models were used to investigate the repeatedly collected questionnaires. In general, each model was formulated twice: first to estimate a mean (effect of covariates on) QoL irrespective of time and then to investigate the evolution of the (effect of covariates on) QoL over time. For all LME models, random (subject-specific) slopes were not deemed appropriate using likelihood ratio tests and thus only included subject-specific intercepts. Marginal and conditional residual plots were used to check model assumptions. The fixed effects for each of the LME models were as follows. First, the mean EQ-5D-5L and HADS anxiety and depression scores were determined using an intercept-only LME model, with the QoL scales consecutively as the dependent variable, to account for the presence of repeated measurements. Then, the change over time was quantified by including time to end of study as an independent variable in the previous models. To investigate whether the planning of surgery had an influence on health-related QoL, anxiety, and depression, and their evolution over time, the latter model was respectively extended with end of study event status (that is patient did or did not undergo surgery) and its interaction with time as independent variables. Lastly, the effects of age, sex, education level (primary education alone, secondary education, or university/college education), polypharmacy (greater than or equal to 5 different daily medications), family history of AAA (first-degree relative with an AAA), baseline maximum AAA diameter, and aneurysm growth rate were investigated, given their proven or hypothesized impact on patient-reported outcomes^23–25^. As non-cardiovascular co-morbidity was not prospectively collected by this study, polypharmacy was chosen as an encompassing variable to represent both the presence and severity of multimorbidity, while capturing the independent effect of polypharmacy on patient-reported outcomes as well^24^. Aneurysm growth rate was based on repeated CT-based maximum diameter growth and estimated using the patient-specific slopes of a linear random-effects model including time as the random effect. To investigate the effects of the mentioned covariates on QoL, the previous fixed effects of the LME models were again extended to a multivariable model by including these covariates as independent variables, first without their interaction with time and then all with their interaction with time, to respectively calculate their mean effect on QoL and investigate their effect on change in QoL over time. Multicollinearity was checked using variance inflation factors.

To confirm whether the determinants associated with the continuous outcomes also represented differences in possible clinically relevant anxiety or depression (dependent variable), these determinants were used as independent covariates in univariable logistic mixed-effect models, taking event status (surgery *versus* no surgery) at the end of the study into account.

For all tests, *P* < 0.050 was considered statistically significant. The analysis was performed in R (version 4.3.2), using the mice (version 3.16.0), eq5d (version 0.15.0), nlme (version 3.1-161), and lme4 (version 1.1-35.1) packages.

## Results

### Study population

Of the participants, two did not respond to any of the questionnaires, leaving 124 patients available for analysis. The mean(s.d.) age of the patients with an AAA was 72(7.4) years. Of the patients, 112 (90.3%) were male and 100 (80.6%) were living with another person. The majority of the patients (82, 66.1%) had a secondary education, followed by a primary education alone (23, 18.5%) and a university/college education (19, 15.3%). Other patient characteristics are presented in *Table 1*. The median baseline maximum AAA diameter was 46 (i.q.r. 43–49) mm and 34 (27.4%) patients underwent surgery within the follow-up interval.

**Table 1:** Clinical characteristics of included patients; *n* = 124

	Value
Age (years), mean(s.d.)	72(7.39)
Male	112 (90.3)
BMI (kg/m^2^), median (i.q.r.)	26.9 (25.3–29.7)
**Living situation**	
Living with another person	100 (80.6)
Living alone	24 (19.4)
**Education level**	
Primary education alone	23 (18.5)
Secondary education	82 (66.1)
University/college education	19 (15.3)
**Risk factors and co-morbidity**	
Currently smoking	42 (33.9)
Dyslipidaemia	85 (68.5)
Arterial hypertension	93 (75.0)
Diabetes mellitus	28 (22.6)
Chronic obstructive pulmonary disease	25 (20.2)
Chronic kidney disease	26 (21.0)
**Cardiovascular disease**	
Coronary artery disease*	45 (36.3)
Cerebrovascular disease	19 (15.3)
Heart failure	9 (7.3)
Peripheral arterial occlusive disease	30 (24.2)
**Medication**	
Antihypertension medication	93 (75.0)
Antiplatelet medication	87 (70.2)
Lipid lowering medication	97 (78.2)
Polypharmacy (≥5 daily medications)	86 (69.4)
**AAA**	
Family history of AAA†	29 (23.4)
Iliac aneurysm	23 (18.5)
Anatomical AAA classification—infrarenal	118 (95.2)
Baseline maximum AAA diameter (mm), median (i.q.r.)	45.5 (42.75–49.0)

Values are *n* (%) unless otherwise indicated. *At least one of the following: history of myocardial infarction, percutaneous coronary intervention, and coronary artery bypass grafting. †First-degree relative with an abdominal aortic aneurysm, based on anamnestic information. AAA, abdominal aortic aneurysm; i.q.r., interquartile range.

### Response rate

**Fig. 1:**
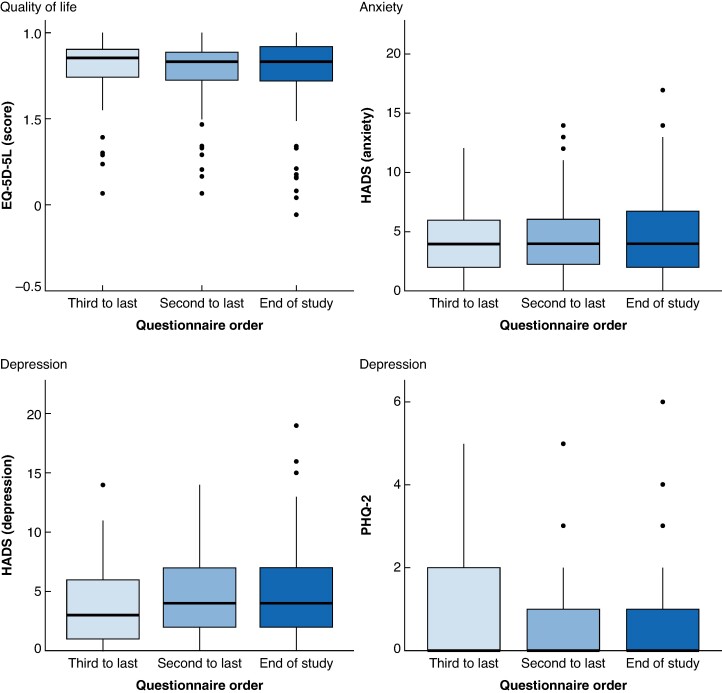
Summarized patient-reported outcomes over time Distribution of patient-reported outcome measures at each moment in time up to the end of the study, which was either the end of follow-up or until abdominal aortic aneurysm surgery/death. EQ-5D-5L, EuroQol five-dimension five-level; HADS, Hospital Anxiety and Depression Scale; PHQ-2, short version of the Patient Health Questionnaire.

At baseline, 123 (99.2%) AAA patients completed the EQ-5D-5L and HADS questionnaires and 122 (98.4%) patients completed the PHQ-2. At the 1-year follow-up, 97 (96.0%) of 101 alive and untreated participants responded to all questionnaires. At the 2-year follow-up, 74 (96%) of 77 alive and untreated participants answered the EQ-5D-5L and HADS questionnaires and 72 (94%) answered the PHQ-2. A summary of the responses over time to each questionnaire is shown in *Fig. 1*.

### Health-related quality of life

**Fig. 2:**
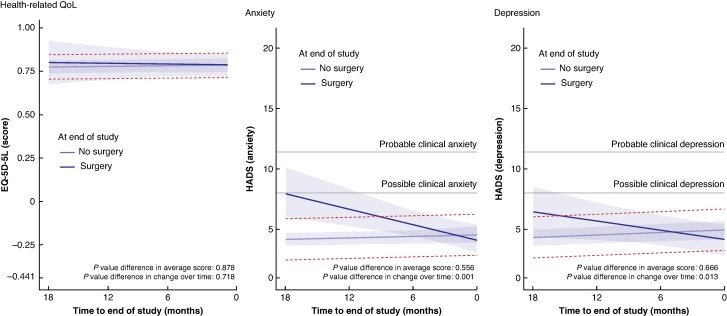
Health-related quality of life, anxiety, and depression over time in patients with an abdominal aortic aneurysm under surveillance Estimated evolution over time, including the 95% confidence intervals, of health-related quality of life, anxiety, and depression in patients with an abdominal aortic aneurysm under surveillance. The horizontal dashed lines represent the interval of the minimal clinically important difference in patient-reported outcomes from the trajectory of the patients who did not undergo surgery. When the trajectory of the patients who underwent surgery lies beyond this interval, it can be concluded that differences in the trajectories are clinically meaningful. For both Hospital Anxiety and Depression Scale subscales, two horizontal continuous lines represent the thresholds where clinical anxiety or depression is possible (Hospital Anxiety and Depression Scale score of 8) and probable (Hospital Anxiety and Depression Scale score of 11). EQ-5D-5L, EuroQol five-dimension five-level; HADS, Hospital Anxiety and Depression Scale.

The mean health-related QoL score of patients with an AAA under surveillance was 0.781 (95% c.i. 0.749 to 0.814) and remained stable over time (mean score change per year 0.001 (95% c.i. −0.001 to 0.002); *P* = 0.460). The distribution of EQ-5D-5L subscores can be found in *Fig. S2*. There were no significant differences between patients who did undergo surgery and patients who did not undergo surgery within the study follow-up interval in mean health-related QoL (mean difference 0.006 (95% c.i. −0.071 to 0.083); *P* = 0.878) or its change over time (mean difference in score change per year −0.016 (95% c.i. −0.103 to 0.071); *P* = 0.718) (*Fig. 2*). At a mean of 75% (95% c.i. 72% to 77%), the EQ-5D-5L VAS showed similar associations over time and with respect to surgery (*Table S1*).

On average, male patients had a 0.188 (95% c.i. 0.078 to 0.297) higher EQ-5D-5L index score, compared with female patients (*P* = 0.001). Additionally, patients taking greater than or equal to five different daily medications had a 0.079 (95% c.i. 0.01 to 0.145) lower EQ-5D-5L score, compared with patients taking fewer medications (*P* = 0.022). While the mean EQ-5D-5L score did not significantly differ according to AAA size, patients had a decreasing EQ-5D-5L score at a rate of 0.025 (95% c.i. 0.002 to 0.048) per year, per 5 mm difference in baseline AAA size. No other determinants were associated with differences in health-related QoL or its change over time (*Table 2*). Only the association between sex and health-related QoL was confirmed by the EQ-5D-5L VAS (*Table S2*).

**Table 2:** Determinants of health-related quality of life, anxiety, and depression in patients with an abdominal aortic aneurysm under surveillance

Determinant	Health-related quality of life			Anxiety			Depression		
	Mean effect		Change in effect over time, *P*	Mean effect		Change in effect over time, *P*	Mean effect		Change in effect over time, *P*
	β (95% c.i.)	*P*		β (95% c.i.)	*P*		β (95% c.i.)	*P*	
Surgery at end of study	−0.014 (−0.064,0.092)	0.728	0.943	0.0 (−1.1,1.2)	0.938	0.001*	−0.4 (−1.8,1.1)	0.617	0.016*
Age (/year)	−0.003 (−0.008,0.001)	0.136	0.849	0.0 (−0.1,0.1)	0.797	0.758	0.1 (0.0,0.1)	0.220	0.940
Male	0.188 (0.078,0.297)	0.001*	0.445	−1.8 (−3.4,−0.2)	0.036*	0.886	0.7 (−1.3,2.7)	0.507	0.897
Secondary education (*versus* primary education alone)	0.002 (−0.083,0.087)	0.960	0.412	−0.6 (−1.9,0.6)	0.337	0.001*	−1.8 (−3.4,−0.2)	0.027*	0.116
University/college education (*versus* primary education alone)	0.003 (−0.102,0.109)	0.950	0.978	−0.4 (−1.9,1.1)	0.600	0.419	−0.3 (−2.2,1.7)	0.777	0.868
Polypharmacy	−0.079 (−0.145,−0.013)	0.022*	0.564	0.4 (−0.6,1.3)	0.473	0.948	0.3 (−1.0,1.5)	0.689	0.263
Family history of AAA	−0.013 (−0.087,0.060)	0.727	0.418	1.3 (0.2,2.4)	0.021*	0.512	0.1 (−1.3,1.4)	0.925	0.689
Baseline maximum AAA diameter (/5 mm)	−0.036 (−0.075,0.005)	0.090	0.042*	0.6 (0.0,1.2)	0.042*	0.425	0.4 (−0.3,1.2)	0.260	0.405
Estimated annual growth rate (/mm/year)	−0.002 (−0.023,0.020)	0.878	0.896	0.0 (−0.3,0.3)	0.921	0.556	−0.1 (−0.5,0.3)	0.474	0.094

To examine the associations of determinants with health-related quality of life (EuroQol 5-dimension 5-level index score) and anxiety and depression (Hospital Anxiety and Depression Scale score) in patients with an abdominal aortic aneurysm currently under surveillance, regression analysis using linear mixed-effects models was performed. First, the mean effect of each determinant was investigated using multivariable mixed-effects models that did not include any interaction terms between time and the determinants. Then, to investigate whether any of these determinants were associated with a change in quality of life, anxiety, or depression over time, the same multivariable linear mixed-effects models were constructed, now including an interaction between each determinant and time. Results are presented as the mean effect, expressed as difference in EuroQol five-dimension five-level index score or points difference in Hospital Anxiety and Depression Scale anxiety or depression score, with 95% confidence interval, for either the presence of the determinant compared with its absence or a reference category for categorical variables or a one unit increase for continuous variables. *Statistically significant. β, regression coefficient; AAA, abdominal aortic aneurysm.

### Anxiety

The mean HADS anxiety score was 4.4 (95% c.i. 3.9 to 4.9) points, which was stable over time (mean points change per year 0.2 (95% c.i. −0.2 to 0.5); *P* = 0.378). Although patients who had undergone surgery by the end of the study did not have a significantly different mean score (0.3 (95% c.i. −0.8 to 1.5) points; *P* = 0.556), their anxiety declined over time with a mean of 2.8 (95% c.i. 1.1 to 4.6) points/year more towards the moment of surgery (*P* = 0.001), compared with patients who did not undergo surgery (*Fig. 2*).

**Fig. 3:**
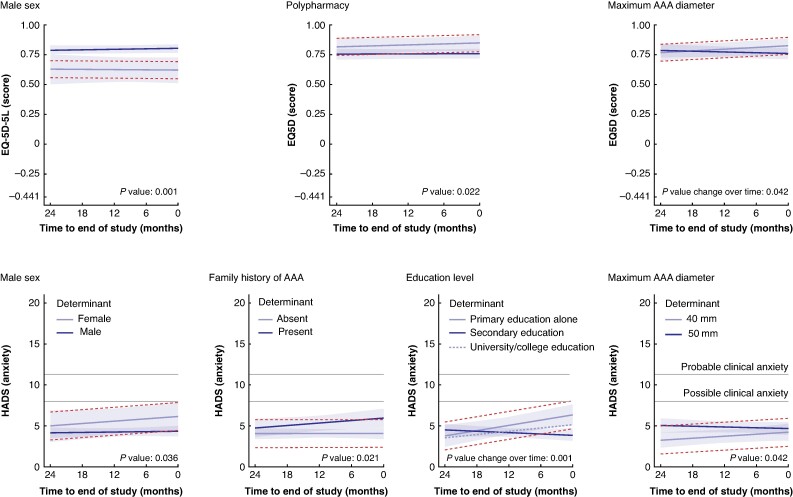
Determinants of patient-reported outcomes in patients with an abdominal aortic aneurysm under surveillance The effects of several determinants on the course of health-related quality of life (EuroQol 5-dimension 5-level index score) and anxiety (Hospital Anxiety and Depression Scale) in patients with an abdominal aortic aneurysm (*y*-axis) over time (*x*-axis) is shown. The full range of possible values is shown on the *y*-axis. The horizontal dashed lines represent the interval of the minimal clinically important difference in patient-reported outcomes from the trajectory of the reference group. When the trajectory of the risk group lies beyond this interval, it can be concluded that differences in the trajectories are clinically meaningful. For the Hospital Anxiety and Depression Scale anxiety subscale, two horizontal continuous lines represent the thresholds where clinical anxiety is possible (Hospital Anxiety and Depression Scale score of 8) and probable (Hospital Anxiety and Depression Scale score of 11). EQ-5D-5L, EuroQol five-dimension five-level; HADS, Hospital Anxiety and Depression Scale.

On average, male patients with an AAA had a 1.8 (95% c.i. 0.2 to 3.4) points lower mean anxiety score, compared with female patients (*P* = 0.036). Additionally, patients with a first-degree relative with an AAA had a significant 1.3 (95% c.i. 0.2 to 2.4) points higher mean anxiety score, compared with patients without a first-degree relative with an AAA (*P* = 0.021) (*Fig. 3*). Whereas anxiety in patients with a secondary education did not significantly change over time, anxiety in patients with a primary education alone significantly increased with a mean of 1.6 (95% c.i. 0.7 to 2.5) points/year more (*P* = 0.001) (*Fig. 3*). Lastly, patients with larger AAAs had a 0.6 (95% c.i. 0.0 to 1.2) points higher mean anxiety score per 5 mm difference in baseline AAA size (*P* = 0.042) (*Fig. 3*). Other factors did not show significant associations with the mean anxiety or change in anxiety over time (*Table 2*).

Of the 112 male patients, 26 (23.2%) had at some point during their follow-up possible clinically relevant anxiety, whereas this was the case for 6 of the 12 female patients. This translated into a trend towards a three-fold lower risk of possible clinical anxiety for male, compared with female, patients throughout follow-up (OR 0.31 (95% c.i. 0.10 to 1.02); *P* = 0.055). Of 29 patients with a family history of AAA, 14 patients had, at some point during the study, possible clinically relevant anxiety, compared with 18 of 95 (19%) patients without a family history of AAA. Correspondingly, patients with a family history of AAA had a three-fold higher risk (OR 3.62 (95% c.i. 1.58 to 8.30); *P* = 0.002) of possible clinically relevant anxiety, irrespective of whether patients proceeded to surgery. Patients with a secondary education had a lower risk of possible clinically relevant anxiety (15 of 82, 18%) throughout the study, compared with patients with a different education level (17 of 42) (OR_secondary *versus* other_ 0.40 (95% c.i. 0.17 to 0.90); *P* = 0.026). Lastly, baseline AAA size was not associated with the risk of possible clinically relevant anxiety (OR per 5 mm maximum AAA diameter difference 1.13 (95% c.i. 0.67 to 1.89); *P* = 0.654).

### Depression

The mean HADS depression score was 4.6 (95% c.i. 4.0 to 5.2) points, which increased at a mean rate of 0.3 (95% c.i. 0.0 to 0.7) points/year (*P* = 0.026). There was no significant difference in mean depression scores between patients who did undergo surgery and patients who did not undergo surgery (mean difference −0.3 (95% c.i. −1.7 to 1.1) points; *P* = 0.666). Similar to the results pertaining to anxiety, patients who underwent surgery within the study interval had decreasing depression scores at a mean rate of 2.0 (95% c.i. 0.4 to 3.6) points/year more, compared with patients who did not undergo surgery (*P* = 0.013) (*Fig. 2*).

Patients with a secondary education, compared with a primary education alone, had a mean of 1.8 (95% c.i. 0.2 to 3.4) points lower depression scores (*P* = 0.027). No other determinants showed significant associations with signs of depression on average or with changes in signs of depression over time (*Table 2*).

Correspondingly, independently of whether patients proceeded to surgery, patients with a secondary education had an almost seven-fold lower risk of experiencing possible clinical depression (16 of 82, 20%), compared with patients with a different education level (18 of 42) (OR 0.15 (95% c.i. 0.03 to 0.75); *P* = 0.021). Using the PHQ-2, there was no indication of an increased risk of depression according to education level (OR_secondary *versus* other_ 0.50 (95% c.i. 0.17 to 1.47); *P* = 0.207).

## Discussion

Health-related QoL, anxiety, and depression were serially measured in 124 patients with an AAA under surveillance. Overall, health-related QoL, anxiety, and depression do not show clinically meaningful changes over time in patients with an AAA under surveillance. Approaching surgery, however, patients experience an important decrease in anxiety and depression scores. Female, compared with male, patients had a lower health-related QoL score, a higher anxiety score, and a higher risk of possible clinical anxiety. Patients taking greater than or equal to five different daily medications, compared with patients taking fewer medications, had a lower health-related QoL. The mean anxiety scores were higher in patients with a first-degree relative with an AAA, corresponding to a three-fold higher risk of experiencing possible clinical anxiety. While the mean anxiety scores did not differ for patients with an AAA with a primary education alone, compared with a secondary education, the anxiety levels of the patients with a primary education alone increased over time. A similar association was observed between education level and signs of depression, with a corresponding seven-fold increased risk of possible clinical depression. However, the latter could not be confirmed by the PHQ-2. Lastly, patients with larger AAAs showed a slight decrease in health-related QoL over time, as well as a tendency to report higher anxiety scores, but not to the extent that this translated to a higher risk of clinical anxiety.

This study offers several advantages over previous research. First, a prospective observational cohort study was conducted, serially measuring patient-reported outcomes over a 2-year interval with a high response rate. This design enabled investigation of changes in QoL over time and assessment of the impact of surveillance in patients with an AAA. Moreover, by incorporating three different questionnaires, a comprehensive evaluation of a patient’s experience was obtained. Additionally, the use of mixed modelling enabled the study to account for informative missing information and individual variability, and to explore the temporal patterns of patient-reported outcomes and their determinants.

On average, the included patients with an AAA had a health-related QoL score that was slightly lower than a previously reported country-specific mean(s.d.) health-related QoL score for the general population of the same age (0.852(0.148))^26^. Although a Swedish general population study reported lower median HADS anxiety and depression scores (2 (i.q.r. 1–5) and 3 (i.q.r. 1–5) respectively)^27^, HADS anxiety and depression scores in the present study did not differ from those previously reported for the Dutch general population of a slightly younger age (median of 4 (i.q.r. 2–7) and 2 (i.q.r. 1–3) respectively)^28,29^. Overall, the patients with an AAA in the present study had mean(s.d.) HADS anxiety and depression scores comparable to those of patients with cancer (5.78(3.90) and 5.41(3.75) respectively)^30^, although depression scores in the present study were higher than those of Dutch patients with newly diagnosed head and neck cancer in a previous study (median depression score of 3 (i.q.r. 1–6))^31^. However, it remains important to note that the mean scores in the patients with an AAA in the present study clearly lie below the median HADS score of 11 in patients with generalized anxiety disorder^32^ and below the mean HADS score of 8 in patients with major depressive disorder^33,34^. Nevertheless, certain subgroups are at risk of a worse disease experience.

Patients who underwent surgery by the end of the study initially had more signs of anxiety and depression, compared with patients with small AAAs who did not require surgery during the study interval. Additionally, the initially higher anxiety and depression scores in these patients had decreased at their last measurement before the procedure. As most patients are aware of the surgical threshold and the association between size and rupture risk^23^, and as a higher perceived chance of rupture is associated with worse emotional QoL^15^, the initial higher anxiety levels in patients with AAAs approaching the surgical threshold might be attributable to the ‘ticking time bomb’ metaphor, frequently associated with aneurysms^5^. It is hypothesized that planning a procedure, and thus resolution of the issue in the following months, might alleviate some of the anxiety. Although the present results may suggest such an association, the data only allowed investigation of linear trends over time. Rather, to substantiate this hypothesis, (repeated) anxiety measurements shortly before and after the decision to move towards surgery would be required.

Similarly, it was found that health-related QoL decreased over time and that there were on average more signs of anxiety in patients with larger aneurysms, compared with patients with smaller aneurysms. As stated above, this is in line with previous findings underlining patient awareness of aneurysm size and rupture risk^23^ and the negative impact of perceived rupture risk on emotional QoL^15^. It is important to note that clinically meaningful differences were only observed between patients with large differences in AAA size. Additionally, no association was found between aneurysm growth rate and patient-reported outcomes, but these findings might relate to the fact that patients are not directly aware of the growth rate of their aneurysm, contrary to the size of their aneurysm.

Sex differences in a person’s experience of health have been described, both in healthy individuals and patients with chronic illnesses^35,36^. Similar to these previous records, female AAA patients had a worse mean health-related QoL, but a similar course over time, compared with male patients. Second, polypharmacy has been put forward as an independent risk factor for a worse health-related QoL in patients with cardiovascular risk factors^24^. The present results confirm and extend these findings.

It is known that other somewhat comparable hereditary diseases, such as intracranial aneurysms, entail an increased risk of depressive mood^37^. Despite the recognized but unspecified hereditary trait in AAA disease^38,39^, no studies have been published on the effects of family history on patient-reported outcomes of AAA surveillance or screening. This lack of evidence regarding AAA surveillance was addressed by the present study, which characterized patients with a first-degree relative with an AAA as having higher overall anxiety levels and a three-fold higher risk of possible clinically relevant anxiety. Given that the mean difference between patients with and without a family history of AAA was smaller than the MCID and given that the mean HADS scores were far below the ‘clinical anxiety threshold’ of eight, it is more likely that a subgroup of patients with such a family history are at risk of clinical anxiety, rather than this finding being merely due to dichotomization of anxiety alone. A previous study in brothers of patients who underwent an endovascular aneurysm repair did not find a negative impact on QoL due to AAA screening, but did not use questionnaires specifically designed to evaluate anxiety^39^. Therefore, the effect of screening in this high-risk group will be investigated more elaborately by the DAAAD study. This ongoing trial, aimed at investigating the feasibility of targeted screening of first-degree relatives of patients with an AAA using national registries, will characterize patient experience related to the hereditary AAA trait using the same questionnaires that were used in the present study^25^.

Patients with an AAA with a primary education alone had increasing anxiety levels and on average more signs of depression. This might originate from the knowledge gap regarding AAA of participants with a lower education^23^. Although differences in anxiety levels have been shown to flatten at higher education levels^40^, the participants with a university/college education in the present study had higher anxiety levels, compared with those with a secondary education. Contrary to reports where educational attainment is related to singular measurements of anxiety and depression^41^, the present divergent observation was also noted by a previous study investigating how anxiety in patients with an AAA undergoing surgery could be reduced using an eHealth tool^42^. Second, patients with a lower education had an increased risk of depression, which was previously not found in a study in patients with an AAA planned for intervention^43^. Although the more fundamental PHQ-2 tool did not confirm differences in major depressive signs, the confirmatory depression and anxiety findings are not surprising, as, despite a few marked differences in their pathophysiology, these disorders have overlapping characteristics and often coincide^44^. As anxiety might be more easily tackled by targeted interventions^42^, and as it usually precedes depressed moods^44^, anxiety might be the primary target to improve patient-reported outcomes in patients with an AAA under surveillance.

Physicians should be aware of the impact of planning and performing an AAA intervention on anxiety and depression around this interval. All the more as perioperative anxiety can be addressed by an additional consultation and information^42^. Alongside consultation and information tools, cognitive behavioural therapy (CBT) may also be beneficial to reduce anxiety and/or depression in patients under a ‘watchful waiting’ surveillance strategy^45^. Digital applications that incorporate techniques from CBT are currently being explored as an easily accessible alternative to CBT to reduce anxiety and depression in a variety of conditions^46,47^. Knowledge on specific groups at risk of overall worse, or worsening progression of, patient-reported outcomes and understanding which specific domains are affected allow for a tailored approach throughout surveillance. Future research should clarify the place of potential anxiety-reducing strategies in patients with progressive AAAs, a family history of AAA, or a lower education, as well as how depressive signs could be ameliorated or prevented in the latter group.

Several limitations of the present study should be acknowledged. The focus on a single geographical region might limit the broader applicability of the findings, as patient-reported outcomes depend on cultural differences. Second, as the participants in the present study are enrolled in an observational cohort study, requiring some level of participation and motivation, the results might not fully reflect the whole group of patients with an AAA. Similarly, although the questionnaires and the data collection process were designed to minimize response biases, a social desirability bias cannot be ruled out in the patients’ responses. Lastly, these questionnaires were developed to quantify distinct aspects and thus should not be interpreted as covering the full experience of a patient.

On average, health-related QoL, depression, and anxiety remain stable over time in patients with an AAA under surveillance. With surgery approaching, patients experience a decrease in signs of anxiety and depression. As patients with a family history of AAA or a primary education alone experience more anxiety and/or depression, these subgroups might require a more tailored patient approach throughout surveillance.

## Supplementary Material

zrae150_Supplementary_Data

## Data Availability

Anonymized data and code that support the findings of this study will be made available by the corresponding author to other researchers for purposes of reproducing the results upon reasonable request and in accordance with a data-sharing agreement.
