# Supplementary material for: Evolution of quality of life, anxiety, and depression over time in patients with an abdominal aortic aneurysm approaching the surgical threshold
Source: BJS Open. 2025 Jan 10;9(1):zrae150. doi: 10.1093/bjsopen/zrae150 (PMC11720167; doi:10.1093/bjsopen/zrae150)
Supplement: zrae150_Supplementary_Data [file zrae150_supplementary_data.docx]

**Evolution of Quality of Life, Anxiety and Depression over Time in Patients with Abdominal Aortic Aneurysm Approaching the Surgical Threshold**

Authors: Alexander Vanmaele, MD^a,b,*^ and Petros Branidis, MD^a,b,*^, Maria Karamanidou, MD^a,b^, Elke Bouwens, MD, PhD^a,b,c^, Sanne E Hoeks, PhD^c^, Jorg L de Bruin, MD, PhD^b^, Sander ten Raa, MD^b^, K. Martijn Akkerhuis, MD, PhD^a^, Felix van Lier, MD, PhD^c^, Ricardo PJ Budde, MD, PhD^d^, Bram Fioole, MD, PhD^e^, Hence JM Verhagen, MD, PhD^b^, Eric Boersma, PhD^a,*^ and Isabella Kardys, MD, PhD^a,*^

^a^ Department of Cardiology, Thorax Centre, Cardiovascular Institute, Erasmus MC, Rotterdam, the Netherlands.

^b^ Department of Vascular Surgery, Erasmus MC, Rotterdam, the Netherlands.

^c^ Department of Anesthesiology, Erasmus MC, Rotterdam, the Netherlands.

^d^ Department of Radiology and Nuclear Medicine, Erasmus MC, Rotterdam, the Netherlands.

^e^ Department of Vascular Surgery, Maasstad Hospital, Rotterdam, the Netherlands.

* Both authors contributed equally to this work.

**Corresponding author:**

Prof. dr. I. Kardys

Erasmus MC, University Medical Center Rotterdam

Department of Cardiology, room Na-316

P.O. Box 2040

3000 CA Rotterdam, the Netherlands

Phone: +31 6 50032051 E-mail: [i.kardys@erasmusmc.nl](mailto:i.kardys@erasmusmc.nl)

**Supplementary Materials - Index**

| **Supplementary Figures and Tables** |  |
| --- | --- |
| Supplementary Figure S1 | *Page 3* |
| Supplementary Figure S2 | *Page 4* |
| Supplementary Table S1 | *Page 5* |
| Supplementary Table S2 | *Page 5* |

**Supplementary Figures and Tables**

**
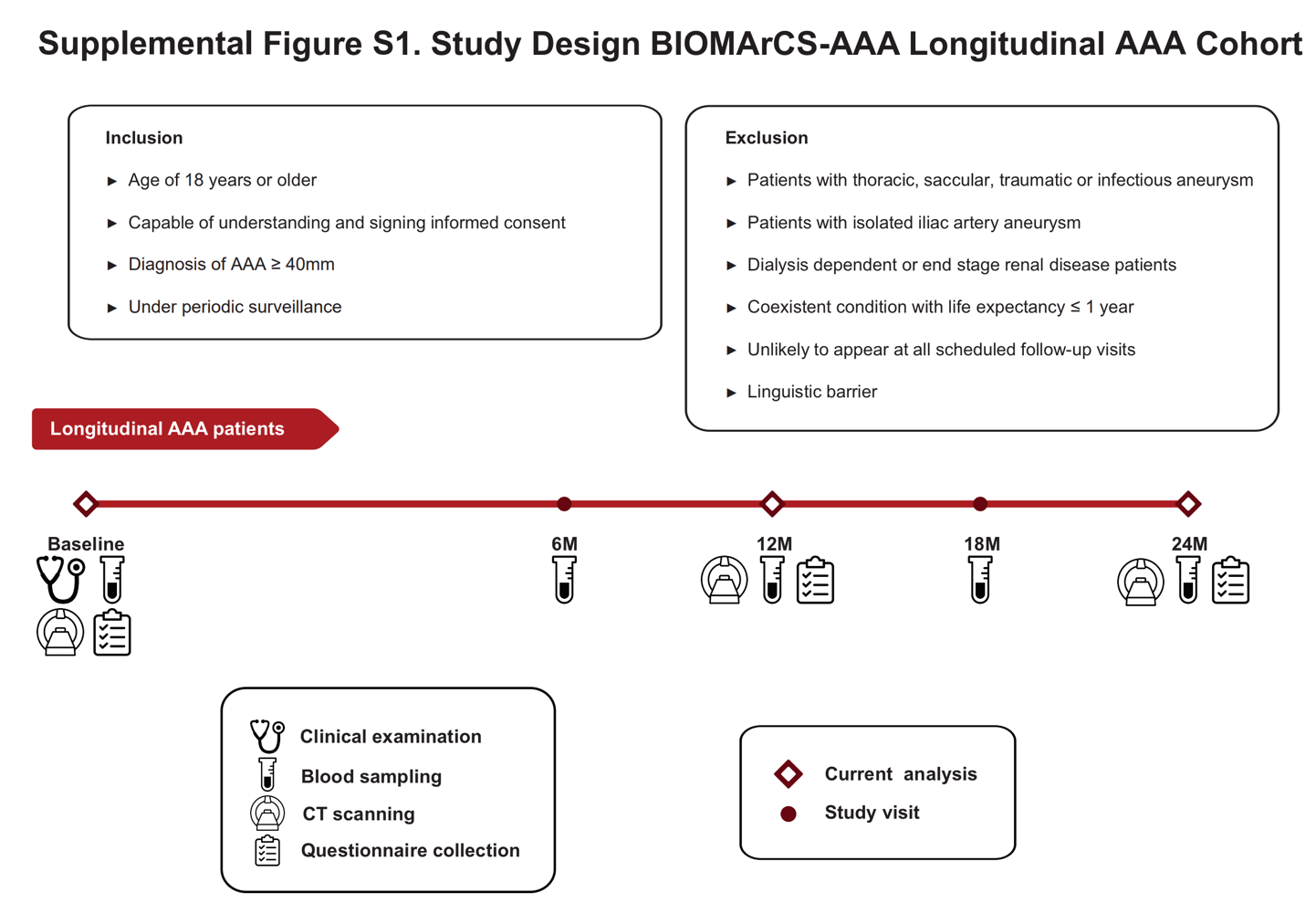
**

**
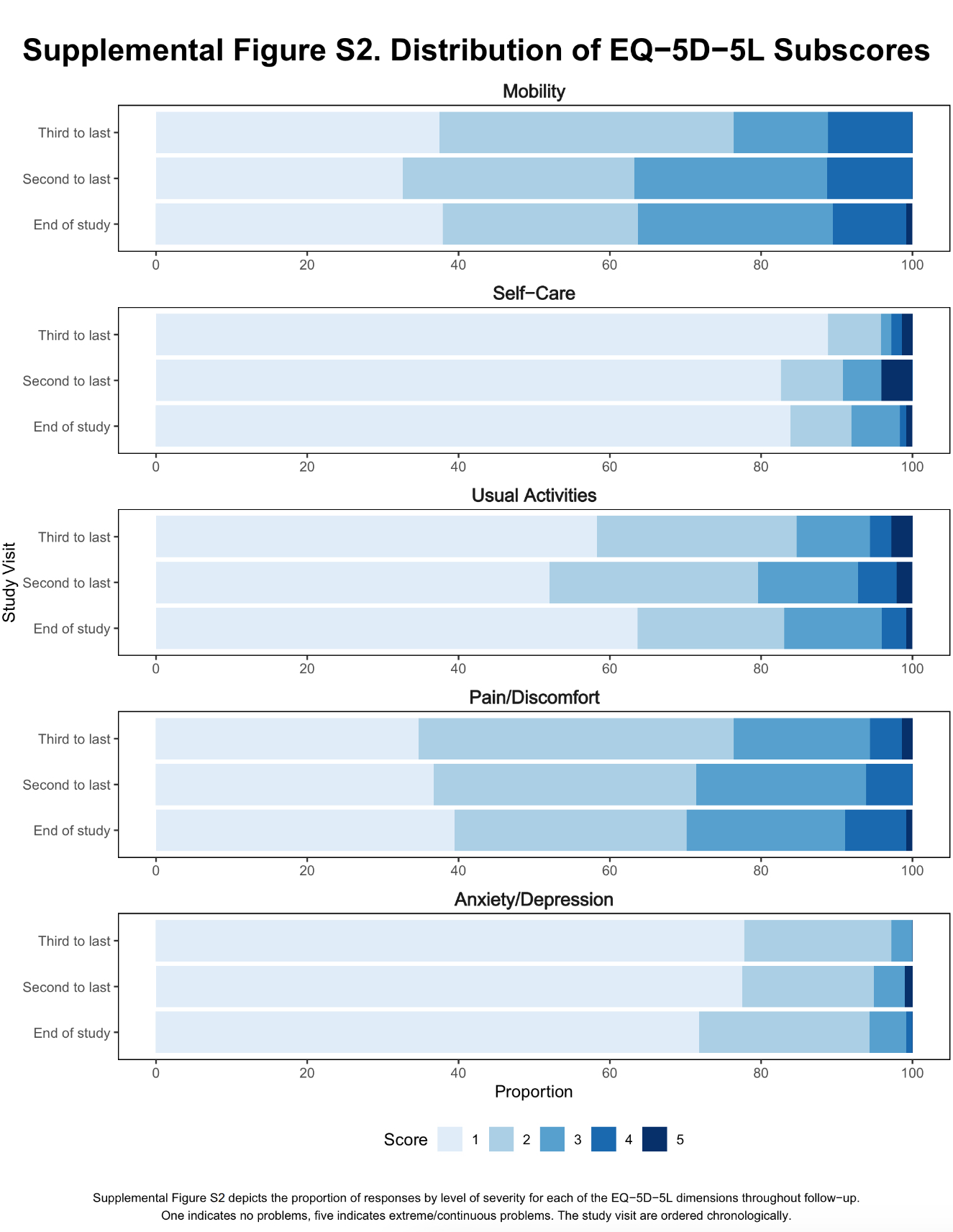
**

**Supplementary Table S1. The Evolution of Health-Related Quality of Life in Patients with AAA under Surveillance.**

| **Supplementary Table S1. The Evolution of Health-Related Quality of Life in Patients with AAA under Surveillance.** | | | | | |
| --- | --- | --- | --- | --- | --- |
|  | Average | | Change over time (/year) | | |
|  | Mean | 95% CI | Mean | 95% CI | p-value |
| All participants | 75 | (72, 77) | -1 | (-2, 1) | 0.409 |
| Patients undergoing surgery | 77 | (72, 82) | 2 | (-6, 10) | 0.541 |
| To examine the evolution of health-related quality of life (EQ-5D-5L visual analogue score, range: 0-100), regression analysis using linear mixed effects models was performed. First, the average VAS in the full study population was investigated by an intercept only, followed by a model including surgical status at the end of the study as an independent variable to estimate the mean VAS in this subgroup. Secondly, time (and its interaction with surgical status) was included in both models to estimate the change in VAS over time. | | | | | |
|  | | | | | |
| Results are presented as the mean (change in) EQ-5D-5L visual analogue score (expressed as percentage), with 95% confidence interval. Significant coefficients are presented in **underlined bold**. | | | | | |

**Supplementary Table S2. Determinants of Health-Related Quality of Life in Patients with AAA under Surveillance.**

| **Supplementary Table S2. Determinants of Health-Related Quality of Life in Patients with AAA under Surveillance.** | | | | |
| --- | --- | --- | --- | --- |
| Determinant | **Health-related Quality of Life (VAS)** | | | |
|  | Average effect | | | Change in effect over time |
|  | β | 95% CI | p-value | p-value |
| Surgery at End of Study | 4 | (-3, 10) | 0.269 | 0.728 |
| Age (/year) | 0 | (-1, 0) | 0.149 | 0.400 |
| Male Sex | **9** | **( 1, 18)** | **0.035** | 0.794 |
| Secondary Education (vs. Primary Education) | 5 | (-2, 11) | 0.176 | 0.350 |
| Higher Education (vs. Primary Education) | 1 | (-7, 10) | 0.764 | 0.475 |
| Polypharmacy | -1 | (-7, 4) | 0.579 | 0.311 |
| Familial AAA History | -2 | (-7, 4) | 0.604 | 0.528 |
| Baseline maximum AAA diameter (/5mm) | -1 | (-4, 2) | 0.478 | 0.693 |
| Estimated Annual Growth Rate (/mm/year) | 0 | (-2, 2) | 0.986 | 0.255 |
| To examine the associations of determinants with quality of life (EQ-5D-5L visual analogue score, range: 0-100), regression analysis using linear mixed effects models was performed. First, the average effect of each determinant was investigated by multivariable mixed effects models which did not include any interaction terms between time and the determinants. Then, to investigate whether any of these determinants were associated with a change in quality of life, anxiety or depression over time, the same multivariable linear mixed effects models were constructed, now including an interaction between each determinant and time. | | | | |
|  | | | | |
| Results are presented as the mean effect, expressed as difference in EQ-5D-5L visual analogue score (expressed as percentage), with 95% confidence interval for either the presence of the determinant compared to its absence or a reference category for categorical variables, or one unit increase for continuous variables. Significant coefficients are presented in **underlined bold**. | | | | |
|  | | | | |
| Abbreviations: AAA: abdominal aortic aneurysm, β: regression coefficient, CI: confidence interval, mm: millimetre. | | | | |
